# Fantastic Frustrated Materials–and Where to Find Them

**DOI:** 10.1021/acscentsci.4c01409

**Published:** 2024-09-12

**Authors:** Christopher R. Wiebe

**Affiliations:** Department of Chemistry, University of Winnipeg, Winnipeg, Manitoba R3B 2E9, Canada

Whether chemists like to admit it or not, topology plays a role in the discovery of new compounds. In fact, chemistry might be defined as the use of topology to understand how new atoms and molecules can be combined into ever more complex structures. Some modern examples of this include the synthesis of metal–organic frameworks (MOFs),^1^ the science of protein folding,^2^ and the design of altermagnets.^3^ Over the past decade, the use of topology has become more prominent in the field of solid state chemistry. Traditionally, nature has been used as a source of inspiration for new materials, using the plethora of well-categorized mineral structures as templates for rational design. However, it can be overwhelming to explore the rich database of known materials. Where does one start to find new magnetic materials with ever increasingly complicated networks of magnetic ions? This is where topology comes in as a valuable tool. A systematic way of classifying structures and narrowing down where to look for certain exotic magnetic states using topology has been lacking–until now, as highlighted in this issue of *ACS Central Science* by the work of Paddison and Cliffe.^4^

Paddison and Cliffe recently investigated various symmetry classes of magnetic systems using a language that chemists can appreciate: The Reticular Chemistry Structural Resources (RCSR) nomenclature.^5^ By breaking down various spin sublattices into different topology classes, they performed Monte Carlo simulations to identify which possible systems—such as the corner-shared tetrahedral pyrochlore lattice or the edge-shared tetrahedral lattice in the face-centered cubic structure—show interesting magnetic ground states (Figure 1). These sublattices are all examples of geometric frustration—where spins are arranged such that conventional long-ranged ordered states are suppressed. The work of Paddison and Cliffe significantly narrows down where chemists should focus their efforts looking for interesting frustrated magnetic states, and in particular, where to look for one of the holy grails of solid state chemistry, the spin liquid. Spin liquids are dynamic but highly correlated systems of spins first predicted by theorists nearly 50 years ago, but very few clean examples have been realized.^6^ Their discovery would not only aid theorists in understanding highly correlated electron systems but also serve as targets for technologically important materials, such as high temperature superconductors, which can be created from spin liquids.^7^

**Figure 1:**
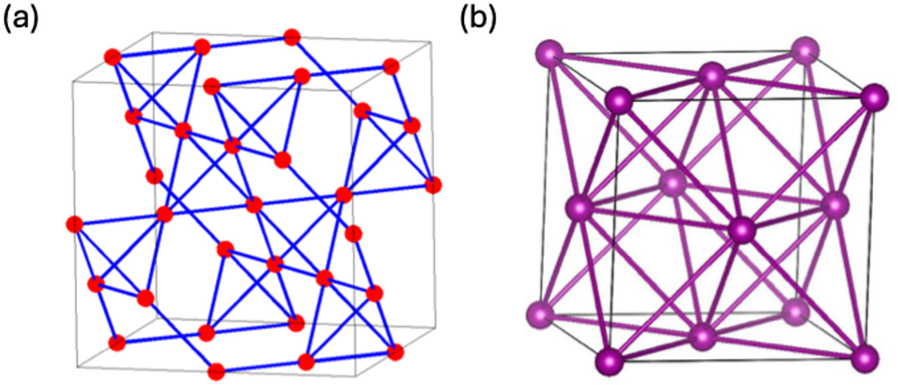
(a) Pyrochlore (**crs**) and (b) face-centered cubic lattices (**fcu**), two of the structure types explored by Paddison and Cliffe in their search for exotic magnetic phases.

There are limits to the analysis by Paddison and Cliffe. Many magnetic systems have significant next nearest neighbor interactions, for example. These more complicated exchange interactions have been left out of the study. As well, the magnetic ground state can be heavily influenced by local crystal field effects. A flavor of this is captured in Paddison and Cliffe’s work by considering local Ising, XY and Heisenberg symmetries; however, a full analysis of ground state stability would also be needed to address the local crystal field and more complicated interactions, such as Dzyaloshinskii-Moriya or antisymmetric exchange interactions.^8,9^ However, this does not take away from the tour de force that this work represents. Nearest neighbor interactions tend to be dominant in many magnetic materials, especially in rare earth f-electron systems with weak exchange. The strength of this work is that the authors have significantly limited which crystal structures and sublattice symmetries can give rise to exotic magnetic states at low temperatures. This will greatly simplify the search for new frustrated materials. In addition, the work shows what characteristic magnetic diffuse neutron scattering profiles are expected in many of these systems at low temperatures. This will make the identification of such states easier, as neutron scattering continues to rise as one of the premier techniques for elucidating magnetic ground states. Chemists will no longer have to sift through the thousands of possible structures to look for novel magnetism. Paddison and Cliffe provide a clear guide for fantastic frustrated materials—and where to find them.

## References

[ref1] Glasby L. T.; Cordiner J. L.; Cole J. C.; Moghadam P. Z. Topological characterization of metal-organic frameworks: a perspective. Chem. Mater. 2024, 10.1021/acs.chemmater.4c00762. PMC11467834 39398380

[ref2] Scalvini B.; Sheikhhassani V.; Mashaghi A. Topological principles of protein folding. Phys. Chem. Chem. Phys. 2021, 23, 21316–21328. 10.1039/D1CP03390E. 34545868

[ref3] Šmejkal L.; Sinova J.; Jungwirth T. Emerging Research Landscape of Altermagnetism. Phys. Rev. X. 2022, 12, 040501 10.1103/PhysRevX.12.040501.

[ref4] Paddison J. A.; Cliffe M. J. Discovering classical spin liquids by topological search of high symmetry nets. ACS The Central Science 2024, 10.1021/acscentsci.4c01020. PMC11503497 39463837

[ref5] O’Keeffe M.; Peskov M. A.; Ramsden S. J.; Yaghi O. M. The Reticular Chemistry Structure Resource (RCSR) Database of, and Symbols for, Crystal Nets. Acc. Chem. Res. 2008, 41, 1782 10.1021/ar800124u. 18834152

[ref6] Lee P. A. From high temperature superconductivity to quantum spin liquid: progress in strong correlation physics. Rep. Prog. Phys. 2008, 71, 012501 10.1088/0034-4885/71/1/012501.

[ref7] Chamorro J. R.; McQueen T. M.; Tran T. T. Chemistry of Quantum Spin Liquids. Chem. Rev. 2021, 121, 2898–2934. 10.1021/acs.chemrev.0c00641. 33156611

[ref8] Dzyaloshinsky I. A thermodynamic theory of “weak” ferromagnetism of antiferromagnets. J. Phys. Chem. Solids 1958, 4, 241–255. 10.1016/0022-3697(58)90076-3.

[ref9] Moriya T. Anisotropic Superexchange Interaction and Weak Ferromagnetism. Phys. Rev. 1960, 120, 91 10.1103/PhysRev.120.91.

